# Flexible bronchoscopy in Ghana: initial experience in a tertiary hospital

**DOI:** 10.11604/pamj.2021.38.298.25833

**Published:** 2021-03-22

**Authors:** Adamu Issaka, Theophilus Adjeso, Iddrisu Baba Yabasin

**Affiliations:** 1 Department of Surgery, Cardiothoracic Surgery Unit, School of Medicine, University for Development Studies, Tamale, Ghana,; 2 Thoracic Surgery Unit, Department of Surgery, Tamale Teaching Hospital, Tamale, Ghana,; 3 Department of Eye, Ear, Nose and Throat, School of Medicine, University for Development Studies, Tamale, Ghana,; 4 Department of Anesthesiology and Intensive Care, School of Medicine, University for Development Studies, Tamale, Ghana

**Keywords:** Bronchoscopy, malignancy, infectious disease, lung diseases, lung cancer, Ghana

## Abstract

**Introduction:**

the use of flexible bronchoscopy in developing countries is limited. We report our initial experience and outcome with the use of flexible bronchoscopy at the Tamale Teaching Hospital in Ghana. This is the first reported case series of flexible bronchoscopy in Ghana.

**Methods:**

a retrospective review of patients who had flexible bronchoscopy from 2017-2019 was analyzed. Patient demographics and outcomes were summarized using frequency distribution and percentages.

**Results:**

we performed flexible bronchoscopies in 33 patients with mean age of 43 years. All patients were symptomatic at the time of presentation with the most common symptoms being chest pain (63.6%), dyspnea (57.6%) and cough (48.5%). The indication for bronchoscopy in most of the cases were suspected malignancy in 16 (48.5%) followed by infection 9 (27.3%), trauma 4 (12.1%) and others 4 (12.1%). We observed abnormal bronchoscopic findings in 25 (75.8%) of the cases with most of the pathologies in the right main bronchus. Twelve patients had toilet bronchoscopy, 6 had biopsy, 5 had no intervention and 4 patients had bronchoalveolar lavage (BAL). Culture and sensitivity results were available for 11 patients, of which 7 patients had negative results. Thirteen (13) malignancies and 11 inflammatory/infectious diseases were diagnosed in this case series. The mean procedure time was 32 minutes with mean hospital stay of 7 days. There was no complication or mortality in our series. **Conclusion:** flexible bronchoscopy is a safe procedure and indispensable in Ghana where there is an increasing incidence of lung diseases.

## Introduction

Bronchoscopy is the use of flexible or rigid endoscopes to view the airways. The flexible bronchoscope has the advantage of being able to be used for viewing the third generation bronchioles. Bronchoscopy has advanced over the past decades from just a diagnostic tool to interventional and endobronchial ultrasound (EBUS) bronchoscopy [1]. It is a commonly performed procedure by pulmonologists, thoracic surgeons, Ear Nose and Throat (ENT) surgeons and anesthetists for different indications, including but not limited to diagnosis, cancer staging, foreign body removal, airway stenting, dilatation, intubation, management of difficult airway and inhalation injuries [2-5].

The use of flexible bronchoscopy in developing countries is however limited, especially in sub-Saharan Africa, where there are few pulmonologists and cardio/thoracic surgeons, which is exacerbated by the lack of needed infrastructure [6-8]. In recent times, Nigeria and South Africa have reported more frequently on their experience and outcomes with flexible bronchoscopy in Africa [7,9-12].

However, in Ghana, very few centers do flexible bronchoscopy relative to the number of centers performing upper gastrointestinal (UGI) endoscopies [13,14]. The Tamale Teaching Hospital (TTH) in the northern part of Ghana is one of the centers with advanced infrastructure and specialists to perform advanced bronchoscopy. The ENT and Cardiothoracic Surgery units routinely perform bronchoscopies. The ENT team usually attends to the emergency cases of foreign body aspiration, whiles the thoracic surgeon does the diagnostic and interventional procedures related to lung pathologies. The Tamale Teaching Hospital and National Cardiothoracic Center in Korle-Bu Teaching Hospital, Accra, are among the first centers to start flexible bronchoscopy in Ghana; however, there is no published report on flexible bronchoscopy in the world literature from Ghana except other unrelated publications to bronchoscopy [3,15,16]. We report our initial experience and outcomes with flexible bronchoscopy in Tamale Teaching Hospital, which is located in the Northern Region of Ghana, Africa. This is a report of a series of flexible bronchoscopy from Ghana.

## Methods

The study was a retrospective review of patients who had flexible bronchoscopy done in the Tamale Teaching Hospital (TTH) from 2017-2019. The hospital is a tertiary facility in the Northern Region of Ghana which serves as the main referral center for hospitals in the 5 northern regions. It has 802 bed capacity with various disciplines. Until 2016, there was no thoracic surgery services in these regions and cases were being referred to the National Cardiothoracic Center in Accra or Komfo Anokye Teaching Hospital in Kumasi for management. The ENT unit of TTH was, however, well established since 2008 and has all the bronchoscopes and human resource that made it easy for bronchoscopy services to be set up.

The Thoracic Surgery Unit has a 10-bed capacity ward running outpatient, inpatient, accident and emergency and theater services to an estimated population of more than 4 million people. The inclusion criteria for the study were all cases of flexible bronchoscopies done within the study period. The data collection excluded cases done using rigid bronchoscopy. Thirty-three cases of flexible bronchoscopy done during this period were included in the study. Patient history, physical examination, laboratory and routine chest X-ray investigations were used in the clinical diagnosis and preoperative planning. Twenty-four patients had a chest computed tomography (CT) scan.

The procedures were done under general anesthesia or conscious sedation in the presence of an anesthetist in the theatre after informed consent is obtained. We commonly use topical lidocaine and intravenous midazolam (occasionally) for conscious sedation. We applied topical lidocaine to the oro-nasopharynx, vocal cords and trachea in conscious sedation anesthesia. Atropine was not routinely given to all patients.Vital signs of patients were monitored before, during and after the procedure. Five biopsy samples were taken in patients with endobronchial tumor.

A fiberoptic bronchoscope (Karl Storz, Tuttlingen, Germany) of different sizes connected to a camera head was used for the flexible bronchoscopy. Data analyzed included patient demographics, indications for bronchoscopy, bronchoscopy findings, duration of procedure, type of intervention, location of pathologies, complications, hospital stay, microbiology, cytology and histopathological findings. The indications for bronchoscopy were grouped into malignancy, infection, trauma and others. The data was collected using Microsoft Excel 2013 and statistical analysis done using the statistical software SPSS version 21 (SPSS Inc., Chicago, IL, USA). Data were presented as frequencies, means and percentages.

## Results

Between 2017 and 2019, we performed 33 flexible bronchoscopies in 33 patients (6 in 2017, 9 in 2018 and 18 in 2019) at the Tamale Teaching Hospital. The mean age of our patients was 43.9 ± 22.8 years with male predominance (n = 20, 61%). The cases were referrals from private and public hospitals, our Accident and Emergency Department, medical and pediatric wards of TTH. Only two of the patients were seen at the thoracic clinic (Table 1). All patients were symptomatic at the time of presentation with the most common symptoms being chest pain (63.6%), dyspnea (57.6%) and cough (48.5%). Five patients had symptoms of dyspnea, chest pain and cough at the time of presentation (Table 1).

**Table 1:** patient demographics

Variables	n (%)
**Sex**	
Male	20 (60.6)
Female	13 (39.4)
**Age (years)**	
≤ 18	4 (12.1)
19 - 70	22 (66.7)
**> 70**	7 (21.2)
**Referral**	
In-hospital wards	17 (51.5)
Accident and Emergency Department	7 (21.2)
District hospitals	4 (12.1)
Private hospitals	3 (9.1)
Thoracic clinic	2 (6.1)
**Presenting symptoms**	
Dyspnea	19 (57.6)
Chest pain	21 (63.6)
Cough	16 (48.5)
Dyspnea, chest pain and cough	5 (15.2)
Others	11 (33.3)
**Previous intervention**	
None	13 (39.4)
Tube thoracostomy	9 (27.3)
Thoracocentesis	4 (12.1)
Biopsy	3 (9.1)
Pulmonary TB treatment	3 (9.1)
Others	5 (15.2)
**Indications**	
Cancer	16 (48.5)
Infection	9 (27.3)
Trauma	4 (12.1)
Others	4 (12.1)
Ventilator dependent diaphragmatic paralysis	1 (3.0)
Trapped lung	1 (3.0)
Catamenial hemopneumothorax	2 (6.0)

Among the Twenty patients who had prior interventions, 13 (39.4%) interventions were pleural related that included thoracocentesis and tube thoracostomy. Three patients had been previously treated for pulmonary tuberculosis and 13 patients had no previous intervention before the bronchoscopy (Table 1). Twenty-four patients (72.7%) had bronchoscopy done under general anesthesia followed by Video Assisted Thoracoscopic Surgery (VATS) or thoracotomy, whiles the remaining were done with conscious sedation.

**Figure 1:**
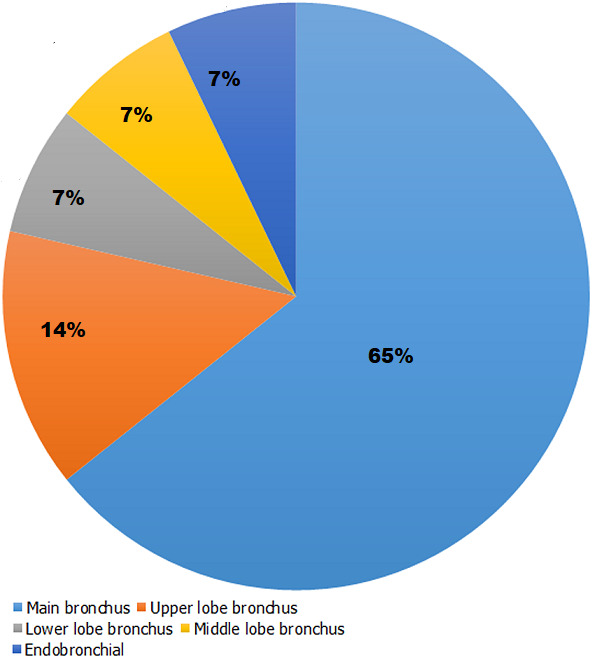
distribution of pathologies in the right endobronchial system

**Figure 2:**
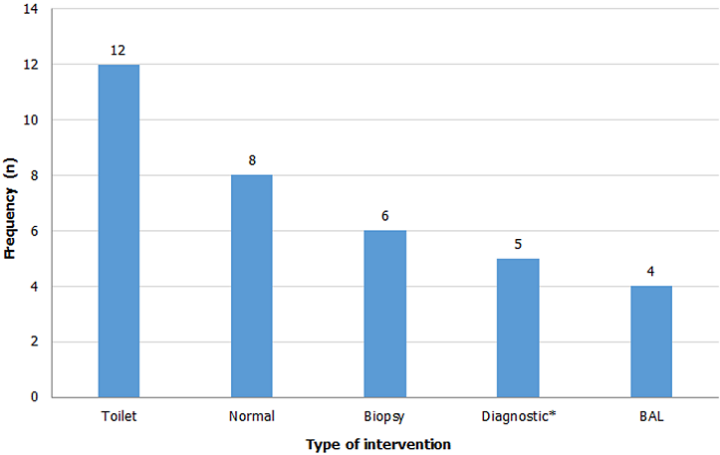
flexible bronchoscopic interventions xNo additional intervention required

The indication for bronchoscopy in most of the cases were suspected malignancy in 16 (48.5%) followed by infection 9 (27.3%), trauma 4 (12.1%) and others 4 (12.1%) (Table 1). We observed abnormal bronchoscopic findings in 25 (75.6%) of the cases. Most of the pathologies were located in the right bronchi 14 (42.4%); with 64.3% found in the right main bronchus as represented in Figure 1. We only found 2 pathologies in the left bronchi. Eight cases had pathologies in both bronchi mainly due to secretions and 1 patient had narrowing of the trachea as a result of compression by an external tumor (Table 2). Normal bronchoscopic findings was recorded in 8 patients where no other intervention was done; 12 patients had secretions that necessitated toilet bronchoscopy; 6 patients with endobronchial lesions had biopsies, 5 patients had pathologies diagnosed on visual examination and 4 patients had bronchoalveolar lavage (BAL) done (Figure 2). The procedures that was commonly done together with flexible bronchoscopy were VATS in 14 patients for biopsy and decortication; and thoracotomy (8 patients) for trauma, lobectomy and decortication.

**Table 2:** bronchoscopic outcomes

Outcomes	n (%)
**Bronchoscopic findings and location**	
Normal findings	8 (24.2)
Pathologic findings:	25 (75.8)
Right bronchi	14 (42.4)
Right and left bronchi	8 (24.2)
Trachea	1 (3.0)
Left bronchi	2 (6.0)
**Cytology/histopathology results**	
Malignancy:	13 (39.4)
Bronchogenic carcinoma	6 (18.2)
Thymoma	2 (6.0)
Angiosarcoma metastasis	1 (3.0)
Hepatocellular carcinoma metastasis	1 (3.0)
Lymphoma	1 (3.0)
Mesothelioma	1 (3.0)
Inflammation/infection	11 (33.3)
Non-specific inflammation	7 (21.2)
Asthma with pneumonia	1 (3.0)
Interstitial lung disease	1 (3.0)
Pneumonia	1 (3.0)
Pulmonary tuberculosis	1 (3.0)
Normal	1 (3.0)
Report not available	1 (3.0)
**Culture and sensitivity/Genexpert results**	
Microorganism/GeneXpert MTB/RIF (-)	7 (21.2)
Microorganism present	4 (12.1)
MTB +	1 (3.0)
Pseudomonas aeruginosa	1 (3.0)
Citrobacter *spp*.	1 (3.0)
Bacterial colonies	1 (3.0)

Culture and sensitivity results were available for 11 patients, of whom 7 patients had negative results for culture and sensitivity and acid-fast bacilli (AFB). Gene Xpert MTB/RIF confirmed pulmonary tuberculosis in one patient who had a negative AFB. The organisms isolated were Pseudomonas aeruginosa and Citrobacter species (Table 2). Twenty-six patients had their samples sent for cytology/histopathology. Of these, 13 had malignancies (of which 6 were bronchogenic carcinomas) and 11 inflammatory/infectious diseases. One patient had a normal histopathology finding as well as 1 patient whose report was not available (Table 2). The mean procedure time for bronchoscopy was 32 minutes with mean hospital stay of 7 days. We did not experience any bronchoscopy-related complication in our series.

## Discussion

During the 3-year period of performing flexible bronchoscopy in TTH in the Northern Region of Ghana, we observed in our study an increasing request and need for flexible bronchoscopy from 6 in 2017 to 18 cases in 2019 mostly for suspected malignancies. Flexible bronchoscopy was done in our institution by applying most of the British Thoracic Society guidelines for diagnostic flexible bronchoscopy in adults where applicable [17]. Over 60% of the cases were male with a mean age of 44 years. Male predominance is also reported in some of the literatures [4,7,18]. Our study included children, hence the relatively younger mean age observed for bronchoscopy. In our study, thoracocentesis and tube thoracostomy were the most commonly done procedures at the referring facility with no definitive diagnosis. This finding is consistent with previous reports in the literature from Africa where the main indications for bronchoscopy included malignancy and pleural effusion which are common indications to perform thoracocentesis and tube thoracostomy [7,9,18-20]. Prior interventions at the referral facility without definitive diagnosis contributed to delays in the timely management of the patients.

All the flexible bronchoscopies were performed in theatre with flexible fiberoptic bronchoscopes instead of a video bronchoscope in a bronchoscopy suite because there is currently no dedicated bronchoscopy unit in the hospital as a result of lack of infrastructure and also most of the cases required surgery under general anesthesia. Because there are usually delays in getting cytology and histopathology reports of samples due to limited pathologists, we usually use multiple diagnostic procedures concurrently to reduce financial burden on patients and also reduce the hospital stay. Flexible bronchoscopy alone was done in 13 patients. There was a wide variation in the indication for flexible bronchoscopy which we grouped into four main categories for easy analysis. Suspected malignancy and infection were the most common indications for bronchoscopy accounting for 75% of all cases, this finding is also consistent with other reports [4,7,9,18]. We however performed flexible bronchoscopy in 4 patients after chest trauma to assess the airway before thoracotomy. This indication is hardly referred to in published literature but is a common one used by thoracic surgeons to assess airway injury or pathology related to the trauma [21].

Out of the 76% abnormal bronchoscopic findings, it was observed that most of the pathologies were in the right endobronchial system specifically the right main bronchus. Pathologies in the right endobronchial system and lung are a common finding in literature, especially in the adult population, mainly because of the angulation and length of the right main bronchus [2,3]. The most commonly diagnosed condition in our patients was cancer of the lungs. There seem to be an increasing trend of lung cancer diagnosed by flexible bronchoscopy in the sub-Saharan African region [4,7,9,18-20]. However, this increase is still limited in comparison to advanced countries due probably to the lack of the disease awareness, availability of diagnostic capabilities and infrastructure including CT scan, flexible bronchoscopy, pathological services and patient´s affordability. Financial constraints has always being of concern in the subregion when it comes to infrastructure development and accessibility of patients as previously reported [4,6,22,23]. In-hospital ward referrals were usually done when patient is not responding to initial treatment or presents with complications. The delays with external referrals were usually due to financial constraints and lack of awareness about expert services in the referral facilities. The delays negatively affected the management outcome of especially cancer patients who presented with advanced disease, hence the limited number of curative treatment in our series.

Culture and sensitivity results were negative in most of the cases suspected to have infections. Pulmonary tuberculosis was diagnosed in only one patient with Gene Xpert MTB/RIF when AFB was negative. Pulmonary tuberculosis is a common lung pathology in the subregion and treatment is mostly based on clinical judgment. The availability and expertise in flexible bronchoscopy in combination with various microbiological and Gene Xpert MTB/RIF would help yield better results as reported by Barnard and colleagues [9,23]. Bronchoscopy-related complications and mortality is reported to be 1.1% and 0.02% respectively [24]. In our study, we did not experience any bronchoscopy-related complications.

**Limitations:** this study has limitations; it is a retrospective study and comes from a single center with limited sample size. Despite these limitations there are still lessons to be learned from our findings. A prospective study in the future in collaboration with some of the hospitals in the subregion will aid throw light on lung diseases in sub-Saharan Africa.

## Conclusion

Flexible bronchoscopy is a safe procedure and indispensable in Ghana, where there is increasing incidence of lung diseases. Medical doctors need to be trained in flexible bronchoscopy and funding made available for infrastructural development. There is the need for health personnel in district hospitals in Ghana to have an increased awareness of lung diseases, including lung cancers.

### What is known about this topic

- Vast publications on rigid bronchoscopy in Africa;
- Usage of flexible bronchoscopy for diagnostic purposes in limited centers in Africa;
- Nigeria and South Africa have published more on their experience compared to other countries in the sub-region.

### What this study adds

- Appropriate management was delayed in most of the cases due to prior intervention at the referral facilities without definitive diagnosis;
- Abnormal bronchoscopic findings in the right main bronchi was recorded in majority of the patients;
- The most common indication for flexible bronchoscopy was suspected malignancy which we found to be in an advanced stage limiting the chance for curative treatment.
